# Premature Bone Resorption in Vertical Ridge Augmentation: A Systematic Review and Network Meta‐Analysis of Randomised Clinical Trials

**DOI:** 10.1111/clr.14435

**Published:** 2025-03-21

**Authors:** Faisal Faihan Alotaibi, Jacopo Buti, Isabella Rocchietta, Nor Shafina Mohamed Nazari, Rawan Almujaydil, Francesco D'Aiuto

**Affiliations:** ^1^ Unit of Periodontology UCL Eastman Dental Institute London UK; ^2^ Department of Oral and Maxillofacial Surgery and Diagnostic Sciences, College of Dentistry Prince Sattam bin Abdulaziz University Alkharj Saudi Arabia; ^3^ Department of Restorative Dentistry, Faculty of Dentistry Universiti Malaya Kuala Lumpur Malaysia; ^4^ Department of Periodontology, College of Dentistry Qassim University Qassim Saudi Arabia

**Keywords:** complications, network meta‐analysis, premature resorption, ridge augmentation, systematic review

## Abstract

**Objectives:**

To systematically appraise the evidence on premature ridge resorption following various vertical ridge augmentation (VRA) techniques in healthy adult patients undergoing staged VRA procedures. The study aimed to identify VRA techniques resulting in the least premature bone resorption and to rank them using Bayesian Network Meta‐Analysis (NMA).

**Material and Methods:**

Searches were conducted in six databases to identify randomised clinical trials (RCT) comparing staged VRA techniques with a minimum of 3 months follow‐up. Relative premature bone resorption (PBR%) overall (primary) and in sites with uneventful versus complicated healing and need for additional bone grafting (NAG) (secondary) were chosen as outcomes. The risk of bias and certainty in evidence were assessed using Cochrane RoB 2.0 and GRADE tools. Bayesian models estimated treatment effects and rankings.

**Results:**

Ten RCTs, involving 220 participants and 236 defects, were included. Nine RCTs reported mean PBR%, with a range from 6% to 44%, averaging 26%. Seven treatment groups were evaluated: onlay, onlay + barrier, inlay, guided bone regeneration, distraction osteogenesis (DO), tissue expansion + tunnelling (TET), and cortical tenting. Eight RCTs, involving 160 participants and 176 defects, contributed to the NMA. Compared to onlay, all groups had lower mean PBR%. Inlay had the highest probability of being ranked first (Pr = 0.55), followed by DO (Pr = 0.27) and TET (Pr = 0.15). Healing complications significantly increased PBR% (MD 10%, 95% CrI 4.4–15.7).

**Conclusion:**

VRA techniques preserving the periosteum (inlay, DO, and TET) exhibit less PBR compared with other techniques. When techniques involve full flap elevation, clinicians should anticipate volume loss at re‐entry and consider greater grafting volumes to offset PBR.

**Protocol Registration:**

PROSPERO ID: CRD42023394396; https://www.crd.york.ac.uk/prospero/display_record.php?RecordID=394396

## 1 Introduction

Tooth loss results in concomitant loss of supporting bone, which in many cases renders the remaining bone volume inadequate for dental implant placement. Surgical augmentation procedures can be performed to restore missing bone either in simultaneous or staged approaches. The former has its own merits but carries an increased risk of dental implant exposure if the bone graft shrinks or is partially resorbed during the healing period. It is therefore recommended mostly in self‐containing bone defects where healing can be reasonably predicted. A staged approach, on the other hand, could be adopted for bone defects that require augmentation beyond the bony envelope or have vertical components to it (Esposito et al. 2009; Jepsen et al. 2019).

Particulate or block types of bone tissue have been used to restore vertically missing alveolar ridges. With the use of particulate bone, experimental studies have shown that the graft is expected to reduce in size (shrink) to nearly half of its original size unless protected by a barrier membrane (Donos et al. 2002). Non‐resorbable membranes offer greater protection against graft shrinkage for cases with vertical bone gain requirements when compared to resorbable membranes. Nevertheless, these membranes are prone to premature exposure, a fairly common clinical complication that could result in the loss of around 40% of bone gained after vertical bone augmentation (VRA) (Tay et al. 2022). Block grafts are also prone to early resorption, even in the absence of complications, with an average amount of 20%–50%, with the percentage increasing in cases of wound exposure (Chiapasco et al. 2006). Combining bone blocks with particulate bone substitutes and/or barrier membranes reduces premature resorption but does not completely eliminate it (Wiltfang et al. 2014; Cordaro et al. 2011; Antoun et al. 2001).

Premature bone graft resorption during the healing phase might not be a serious clinical problem if the defect can be readily overbuilt at the first surgery stage or compensated with additional grafting at the second. While this is true in some clinical cases, others with high aesthetic demands (i.e., anterior maxillae) will usually benefit from stable and predictable reconstruction and the smallest loss in bone graft height so as to avoid negative impact on the final outcomes (Rocchietta et al. 2018). Additionally, overbuilding or additional grafting denotes the increase in tissue manipulation, the amount of bone harvested/grafted, and cost, all essential factors to be factored into proper treatment planning (Alotaibi et al. 2023). To date, there are no systematic reviews evaluating premature resorption of different types of grafts used in VRA. Hence, we aimed to systematically appraise the evidence on premature ridge resorption following various VRA techniques in patients who underwent staged procedures for dental implant placement. Consequently, we sought to identify the VRA technique that results in the least premature bone resorption (PBR), ensuring the greatest stability of grafted bone height, and to compare and rank these techniques using Bayesian Network Meta‐Analysis (NMA).

## 2 Materials and Methods

### 2.1 Protocol Registration and Eligibility Criteria

The review protocol was registered in the PROSPERO database (CRD42023394396), prepared in line with Cochrane Collaboration guidelines and reported following the PRISMA extension statement for reporting of systematic reviews incorporating network meta‐analyses of health care interventions (PRISMA‐NMA) (Hutton et al. 2015).

The following focused question was developed: “In otherwise healthy adult patients who underwent any vertical alveolar ridge augmentation procedure in a staged approach in order to place dental implants, which technique results in the least premature bone resorption, thus ensuring the greatest stability of grafted bone height?”

### 2.2 Study Eligibility Criteria (in PICOS Format)

#### 2.2.1 (P) Population

Healthy adult patients with vertical alveolar ridge deficiencies and in need of dental implant placement. Participants with systemic conditions known to influence VRA outcomes, such as uncontrolled diabetes, bleeding disorders, severe renal or liver diseases, immunodeficiency, and a history of irradiation or chemotherapy, were not included in this review.

#### 2.2.2 (I) Intervention

Any staged VRA procedure utilizing any type of bone graft.

#### 2.2.3 (C) Comparison

Any staged VRA procedure including, but not limited to, distraction osteogenesis, guided bone regeneration (GBR), onlay grafting, and inlay grafting.

#### 2.2.4 (O) Outcomes

##### 2.2.4.1 Primary Outcome

- Clinical or radiographic vertical premature bone resorption, calculated as the percentage of graft height lost during the period between the first stage surgery and the second.

##### 2.2.4.2 Secondary Outcomes

- Difference in percentage of premature bone resorption (calculated as described in primary outcome) in sites with uneventful healing and sites with healing complications.
- Need for additional bone grafting (either partial or new complete bone grafting) at the time of re‐entry surgery (i.e., time of dental implant placement), calculated as a percentage of the number of cases needing additional grafting of all cases.

#### 2.2.5 (S) Studies Type

Parallel or split‐mouth randomised clinical trials (RCTs) with a minimum of 3 months follow‐up post‐augmentation.

### 2.3 Information Sources and Search Strategies

**FIGURE 1:**
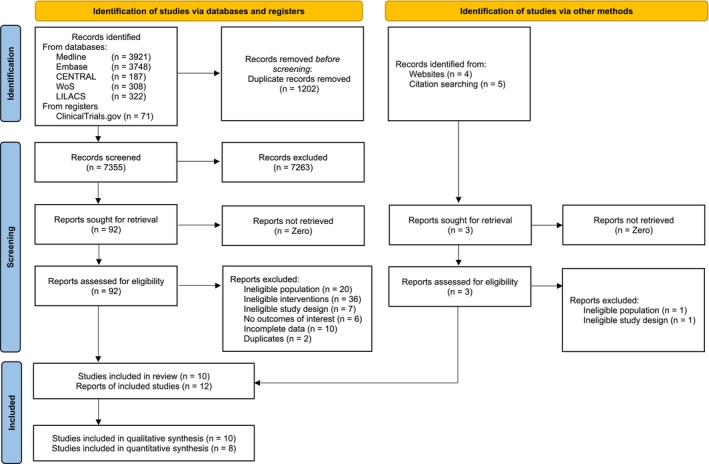
PRISMA flow chart.

Electronic searches up to June 2024 were conducted in six databases: CENTRAL (Cochrane Central Register of Controlled Trials), OVID MEDLINE, EMBASE, Web of Science, LILACS (Latin American & Caribbean Health Sciences Literature) and US National Institutes of Health Ongoing Trials Register (ClinicalTrials.gov) (PRISMA flow‐chart is shown in Figure 1 and databases' search strategies are shown in Appendix 1.1). Articles of any language, but with English titles and/or abstracts, were retrieved. Hand searching was performed in the references of identified studies and reviews, as well as in *Journal of Periodontology*, *Journal of Clinical Periodontology, International Journal of oral and maxillofacial surgery*, *European Journal of Oral Implantology*, *Clinical Oral Implants Research, Clinical Implant Dentistry and Related Research*, *The International Journal of Oral and Maxillofacial Implants*, *International Journal of Oral Implantology*, *and Journal of Oral and Maxillofacial Surgery* for the period between January 2000 and June 2024.

### 2.4 Study Selection and Data Extraction

Screening of titles and abstracts were performed by one reviewer (FFA). In case of uncertainty, a decision on which articles whose full text had to be screened was reached by discussion with the review team. Next, two reviewers (FFA, NSMN) did full‐text screening of the articles for inclusion. The inter‐reviewer reliability (percentage of agreement and kappa correlation coefficient) of the full‐text analysis was calculated. Again, in case of uncertainty, a decision was reached on inclusion or exclusion by discussion with the review team. Next, data extraction forms were developed and piloted on several papers and modified as required before use. Then, data were extracted by two reviewers (FFA, NSMN) followed by verification of a randomly selected sample (50%) of extracted data by two other reviewers (JB, IR). Disagreements were resolved by discussion with a fifth reviewer (FD). Authors of included studies were contacted to provide missing information about study design or outcomes at least on two occasions. The following data were extracted: country and setting, study design, population characteristics (age, sex, general health, smoking, history of periodontitis and location of the alveolar deficiency), intervention details, funding, and conflict of interest. Additionally, information about the primary outcome, including initial vertical bone gain (VBG) (i.e., at stage one surgery), final VBG (i.e., at stage two surgery), and amount of PBR (calculated as the difference in VBG between stage one and stage two surgeries), and secondary outcomes, including amount of PBR in sites with and without healing complications and the need for additional grafting, was extracted into the specially designed extraction forms with conversion made as necessary (i.e., conversion of PBR reported as mm to %).

### 2.5 Data Synthesis

#### 2.5.1 Descriptive Methods

Included studies were summarized, and extracted data were collated in evidence tables to detect any differences in studies' characteristics and to quantify the body of evidence.

### 2.6 Quantitative Methods

Metainsight (v5.2.1) was used for evidence synthesis based on the netmeta package in R statistical software. Bayesian models for meta‐analysis were used, starting with models for pairwise meta‐analysis and then moving on to network meta‐analysis and node‐splitting models (Owen et al. 2019). Both parallel and split‐mouth study designs were included in the analysis and treated the same, as a sensitivity analysis separating the two was not feasible due to the limited number of studies.

#### 2.6.1 Measures of Association

Estimates of treatment effect for direct and indirect evidence from included trials were expressed as mean differences (MD) and 95% credible intervals (CrI) for continuous data and as odds ratios (OR) and 95% CrI for dichotomous outcomes.

#### 2.6.2 Heterogeneity, Inconsistency and Transitivity

Heterogeneity (i.e., between‐trials standard deviation [SD] and 95% CrI) and Inconsistency (i.e., between‐trial differences in the underlying treatment effects between comparisons) were estimated for NMA models (Appendix 1.2).

The assumption of transitivity within the network was assessed by exploring the distribution of patient characteristics, similarity of defects and interventions, and the consistency in study design across comparisons.

To perform the NMA, the most studied treatment (i.e., the most connected treatment group) was considered the primary common comparator, connecting all interventions through direct or indirect comparisons.

#### 2.6.3 Geometry of the Network

Graphical presentations of the evidence base were performed through network plots generated using Metainsight (Owen et al. 2019).

#### 2.6.4 Ranking of Treatments

In addition to relative effects, the Bayesian analysis produced rank probabilities (i.e., the probability for each treatment to obtain each possible rank in terms of their relative effects). Cumulative ranking curves and surfaces under these curves were generated using the Metainsight tool (Owen et al. 2019). The surface under the cumulative ranking (SUCRA) curve is a numeric presentation of the overall ranking and presents a single number associated with each treatment. The higher the SUCRA value (the closer to 100% if expressed in percentage), the higher the likelihood that a therapy is in the top rank, and vice versa.

### 2.7 Regression Analyses

A pair‐wise meta‐analysis to evaluate PBR in sites with uneventful healing versus sites with healing complications was conducted using Metainsight (Owen et al. 2019). Data from RCTs that reported the outcomes at the participant level were used for evidence synthesis.

### 2.8 Assessment of Risk of Bias, Certainty in the Evidence, and Publications Bias

Risk of bias was assessed at the outcome level in all included RCTs using the Cochrane risk of bias (RoB) 2.0 Tool (Sterne et al. 2019). Certainty in the evidence (CiE) was assessed at the outcome level using the Grading of Recommendations Assessment, Development and Evaluation (GRADE) approach (Puhan et al. 2014). Owing to the limited number of studies available for quantitative analysis (< 10 studies), publication bias could not be assessed.

## 3 Results

### 3.1 Study Selection

A total of 8566 records were identified via electronic searches (*n* = 8557) and hand‐searching (*n* = 9). After duplicates removal (*n* = 1202), titles and abstracts of the remaining 7355 records were screened (Figure 1). Following the exclusion of irrelevant titles and abstracts, full texts of 95 publications were screened, leading to the further exclusion of 83 irrelevant articles (Appendix 1.3 shows details of excluded papers with reasons for exclusion). Inter‐rater agreement for study selection, estimated through Cohen's kappa, was 81.8% (kappa = 0.64). Twelve reports of 10 RCTs were included in this review, of which 8 RCTs contributed to the quantitative analysis.

### 3.2 Characteristics of Included Studies

Of the 10 included RCTs, 2 studies had split‐mouth design and 8 had a parallel design (Tables 1 and 2). Three studies compared DO with bone augmentation procedures, and 7 compared two vertical augmentation techniques. The included population consisted of 220 participants, with a total of 236 vertical defects. Among the 10 RCTs (20 intervention arms) a variety of techniques were used for augmentation procedures, including onlay blocks (*n* = 5), inlay blocks (*n* = 3), GBR (*n* = 3), distraction osteogenesis (*n* = 3), tent‐pole (tenting) using cortical shells (*n* = 1), and soft tissue expansion (*n* = 2). Bone materials used for grafting included autogenous (*n* = 7), xenogeneic (*n* = 2), and a mixture of both (*n* = 2). Further information on the characteristics of participants, defects, and interventions in the included studies are provided in the following sections.

**TABLE 1:** Characteristics of included studies and assignment of treatment groups.

		Test			Control				
Study	Design	Technique	Type of bone	NMA group	Technique	Type of bone	NMA group	Age	Outcomes included
	Country	Staging	Barrier (If applicable)		Staging	Barrier (If applicable)		Sex	
	Setting	Sample size			Sample size			Inclusion of smokers	
								Area of the mouth	
Bianchi et al. (2008)	Parallel Italy University of Bologna	Inlay Staged (3–4 months) 5 participants	Monocortical iliac crest bone No membrane	Inlay	Distraction osteogenesis 3–4 months consolidation period 4 participants	NA	DO	45.5 years (mean) 54.4% F ≤ 15 cig./day Posterior mandible	%PBR Complications
Byun et al. (2020))	Parallel Korea 6 university hospitals	TET Staged (4 weeks from TE until grafting then 6 months until implant placement) 23 participants	Xenogeneic particulate bone Collagen membrane	TET	GBR Staged (6 months) 23 participants	Xenogeneic particulate bone d‐PTFE titanium‐reinforced membrane	GBR	57.6 years (mean) 84.6% F Non‐smokers Any area of the mouth	%PBR
Chiapasco et al. (2004))	Parallel Italy University of Milan	GBR Subgroup 1A: simultaneous; Subgroup 1B: staged (6–7 months) 5 participants in subgroup 1B	particulate autogenous bone harvested from ramus, and symphysis if more bone is needed e‐PTFE	GBR	Distraction osteogenesis 2.5–3.5 months until implant placement 10 participants	NA	DO	39.9 years (mean) 57.2% F ≤ 15 cig./day Areas not specified	%PBR
Chiapasco et al. (2007))	Parallel Italy University of Milan	Onlay Staged (4–5 months) 8 participants	Autogenous blocks from mandibular ramus No Barrier	Onlay	Distraction osteogenesis 2.5–3.5 months until implant placement 9 participants	NA	DO	41.2 years (mean) 52.9% F ≤ 15 cig./day Mandible	%PBR
Chiapasco et al. (2013)^a^	Parallel Italy University of Milan	Onlay Staged (4–7 months) 23 participants	Autogenous blocks Resorbable membrane	Onlay	Onlay Staged (4–7 months) 21 participants	Autogenous blocks No barrier	Onlay	53 years (mean) 70.5% F ≤ 20 cig./day Areas not specified	%PBR
Felice et al. (2008)^a^	Split‐mouth Italy University of Bologna	Inlay Staged (4 months) 10 participants	Xenogeneic blocks Resorbable membrane	Inlay	Inlay Staged (4 months) 10 participants	Autogenous blocks Resorbable membrane	Inlay	54 years (mean) 60% F ≤ 15 cig./day Posterior mandible	%PBR
Felice et al. (2009)	Parallel Italy University of Bologna	Inlay Staged (3–4 months) 10 participants	Autogenous blocks Resorbable membrane	Inlay	Onlay Staged (3–4 months) 10 participants	Autogenous blocks Resorbable membrane	Onlay	53.9 years (mean) 70% F NR Posterior mandible	%PBR
Morad and Khojasteh (2013)	Split‐mouth Iran Shahid Beheshti University of Medical Sciences	Tenting Staged (4 months) 6 participants	Cortical plate tented with screws +50:50 autogenous: xenogeneic particulate bone No barrier	CT	Onlay Staged (4 months) 6 participants	Two layers of autogenous blocks covered with particulate bovine bone No barrier	Onlay	55.8 years (mean) 66.6% F NR Posterior mandible	%PBR
Roccuzzo et al. (2007)	Parallel Italy Private practice	Ti‐mesh Staged (4–6 months) 12 participants	Autogenous blocks Ti‐mesh	Onlay + Barrier	Onaly Staged (4–6 months) 12 participants	Autogenous blocks No barrier	Onlay	48.6 years (mean) 66.7% F NR Any area of the mouth	%PBR Complications Need for additional grafting
Shomurodov and Mirkhusanova (2023)	Parallel Uzbekistan Tashkent State Dental Institute	TE then GBR Staged (4 weeks from TE until grafting then 6 months until implant placement) 14 participants	1:1 Autogenous and xenogeneic particulate bone NR	TET	GBR Staged (6 months) 15 participants	1:1 Autogenous and xenogeneic particulate bone NR	GBR	44.8 years 44.7 F Non‐smokers Area not specified	%PBR

Abbreviations: d‐PTFE, dense‐polytetrafluoroethylene; e‐PTFE, expanded‐polytetrafluoroethylene; F, female; GBR, guided bone regeneration; NA, not applicable; NMA, network meta‐analysis; NR, not reported; PBR, premature bone resorption; TE, tissue expansion; TET, tissue expansion + tunnelling; Ti, titanium.

^a^ Compared two interventions within the same NMA group.

**TABLE 2:** Results of individual studies.

Study	Test					Control				
	Technique	Initial VBG	Final VBG	Premature resorption	Need for additional grafting	Technique	Initial VBG	Final VBG	Premature resorption	Need for additional grafting
Bianchi et al. (2008)	Autogenous Inlay blocks	6.07 mm (±0.71)	5.13 mm (±0.57)	0.94 mm (±0.25) 14.7% (±3.6)		Distraction osteogenesis	10.36 mm (±2.96)	8.58 mm (±1.73)	1.98 mm (±1.33) 17.6% (±7.8)	
Byun et al. (2020)	Tissue expansion + tunnelling	5.12 mm (±1.25)	3.55 mm (±1.56)	1.57 mm (±1.04) 30.7% (± 21)		GBR (d‐PTFE + xenogeneic particulate bone	4.22 mm (±1.15)	1.9 mm (±1.58)	2.32 mm (±1.09) 41% (± 19)	
Chiapasco et al. (2004)	GBR (e‐PTFE + particulate autogenous bone)	5.08 mm (±1.06)	3.73 mm (±1.38)	1.35 mm (±0.9) 26.6% (± 18)		Distraction osteogenesis	6.58 mm (±1.38)	5.59 mm (±1.3)	0.37 mm (±0.4) 6% (±7)	
Chiapasco et al. (2007)	Autogenous onlay blocks	5.0 mm (±1.07)	4.4 mm (±1.28)	0.6 mm (±0.7) 12% (±14)		Distraction osteogenesis	5.3 mm (±1.58)	5 mm (±1.61)	0.3 mm (±0.3) 6% (±6)	
Chiapasco et al. (2013)^a^	Autogenous onlay blocks + Resorbable membrane			0.12 mm (±0.32)		Autogenous onlay blocks			0.98 mm (±2.79)	
Felice et al. (2008)^a^	Xenogeneic inlay blocks + Resorbable membrane	6.2 mm (±2.2)	4.52 mm (±1.9)	0.6 mm (±0.4) 10% (±7)		Autogenous inlay blocks + Resorbable membrane	5.1 mm (±1.1)	3.64 mm (±1.1)	1.1 mm (±1.8) 22% (±36)	
Felice et al. (2009)	Autogenous inlay blocks + Resorbable membrane	5.4 mm (±1.1)	4.52 mm (±1.9)	0.72 mm (±0.76) 13.6% (±13.7)		Autogenous onlay blocks + Resorbable membrane	6.52 mm (±1.1)	3.64 mm (±1.1)	2.88 (±1.1) 44.5% (±14.9)	
Morad and Khojasteh (2013)	Tented cortical plate +1:1 autogenous and xenogeneic particulate bone	6.23 mm (±1.24)	5.2 mm (±0.76)	1.75 mm (±1.08) 18.38% (±6.05)		Two layers of autogenous onlay blocks + particulate xenogeneic bone	6.37 mm (±0.71)	4.48 mm (±0.51)	1.17 mm (±0.41) 26.1% (±14.8)	
Roccuzzo et al. (2007)	Autogenous onlay blocks + Ti‐mesh	5.7 mm (±1.5)	4.8 mm (±1.5)	0.9 mm (±0.8) 16.3% (±16)	16.67%	Autogenous onlay blocks	5.5 mm (±1.2)	3.6 mm (±1.4)	1.8 mm (±1.1) 34% (±20)	41.67%
Shomurodov and Mirkhusanova (2023)	Tissue expansion + GBR	5.85 mm (±0.42)		1.78 mm (±0.12) 30.4% (±7.2)		GBR (1:1 autogenous and xenogeneic particulate bone	4.75 mm (±0.16)		2.01 mm (±0.16) 42.3% (±7.5)	

Abbreviations: d‐PTFE, dense‐polytetrafluoroethylene; e‐PTFE, expanded‐polytetrafluoroethylene; GBR, guided bone regeneration; PBR, premature bone resorption; Ti, titanium; VBG, vertical bone gain.

^a^ Did not contribute to quantitative analysis.

### 3.3 Risk of Bias Within Studies and Certainty in the Evidence

Of the 10 RCTs included, eight were judged to have some concerns in relation to risk of bias while 2 were judged to have high risk of bias. The domains “randomisation process” and “measurement of the outcome” were the most serious methodological issues (Appendix 1.4).

Certainty in the evidence ranged from low to very low, with “study limitations” and “imprecision” being the two main reasons for downgrading. The domain “publication bias” could not be assessed due to inapplicability (fewer than 10 studies contributing to the meta‐analysis). No serious issues were observed in relation to inconsistency, indirectness, intransitivity, or incoherence (Table 3).

**TABLE 3:** Premature bone resorption; mean differences (MD) (95% CrI) and certainty in the evidence.

Comparison		Direct		Indirect NMA		NMA	
		MD (95% CrI)	CiE	MD (95% CrI)	CiE	MD (95% CrI)	CiE
Inlay vs.	GBR			−26.53 (−78.63, 23.7)	Low^a^, ^b^	−26.53 (−78.63, 23.7)	Low
	Onlay			−26.59 (−66.37, 10.07)	Low^a^, ^b^	−26.59 (−66.37, 10.07)	Low
	Onlay+Barrier	−30 (−61, 1.2)	Low^a^, ^b^	8.8 (−44, 62)	Low^a^, ^b^	−19.68 (−53.23, 12.76)	Low
	CT			−18.49 (−74.24, 34.31)	Low^a^, ^b^	−18.49 (−74.24, 34.31)	Low
	DO	−3.1 (−34, 27)	Low^a^, ^b^	−42 (−96, 11)	Very low^a^, ^c^	−11.35 (−45.02, 20.66)	Very low
	TET	11.98 (−33.36, 58.05)	Low^a^, ^b^	NE		−15.81 (−74.63, 40.45)	Low
GBR vs.	Onlay			−0.23 (−51.78, 50.75)	Low^a^, ^b^	−0.23 (−51.78, 50.75)	Low
	Onlay+Barrier			6.82 (−47.34, 62.37)	Low^a^, ^b^	6.82 (−47.34, 62.37)	Low
	CT			8.02 (−56.43, 71.94)	Low^a^, ^b^	8.02 (−56.43, 71.94)	Low
	DO	15.07 (−24.06, 54.74)	Low^a^, ^b^	NE		15.07 (−24.06, 54.74)	Low
	TET			10.72 (−16.08, 36.98)	Low^a^, ^b^	10.72 (−16.08, 36.98)	Low
Onlay vs.	Onlay+Barrier	18 (−14, 50)	Low^a^, ^b^	−21 (−74, 32)	Low^a^, ^b^	6.9 (−25.36, 41.79)	Low
	CT	−7.98 (−50.34, 34.84)	Low^a^, ^b^	NE		−7.98 (−46.46, 30.58)	Low
	DO	6 (−25, 37)	Low^a^, ^b^	−45 (−8.6, 98)	Very low^a^, ^c^	15.18 (−17.51, 48.6)	Very low
	TET			10.82 (−46.55, 68.95)	Very low^a^, ^c^	10.82 (−46.55, 68.95)	Very low
Onlay+Barrier vs.	CT			1.14 (−50.54, 50.69)	Low^a^, ^b^	1.14 (−50.54, 50.69)	Low
	DO			8.3 (−30.19, 45.94)	Low^a^, ^b^	8.3 (−30.19, 45.94)	Low
	TET			3.89 (−58.27, 63.71)	Low^a^, ^b^	3.78 (−57.83, 64.1)	Low
CT vs.	DO			7.02 (−42.87, 57.88)	Low^a^, ^b^	7.02 (−42.87, 57.88)	Low
	TET			2.74 (−66.66, 72.64)	Very low^a^, ^c^	2.74 (−66.66, 72.64)	Very low
DO vs.	TET			−4.48 (−52.23, 42.96)	Low^a^, ^b^	−4.48 (−52.23, 42.96)	Low

Abbreviations: CiE, certainty in the evidence; CrI, credible intervals; NE, not estimable (cannot be estimated because the treatment group was not connected in a loop in the evidence network), NMA: network meta‐analysis.

^a^ Downgraded by one due to risk of bias.

^b^ Downgraded by one due to imprecision.

^c^ Downgraded by two due to imprecision.

### 3.4 Descriptive Analysis

#### 3.4.1 Studies' Participants

The included studies analysed a total of 220 participants, with a mean age ranging from 40 to 57 years. The gender distribution showed a predominance of females, ranging from 53% to 85% of participants in nine of the 10 studies. All participants were systemically healthy adults with no reported history of conditions known to impair bone healing. Smokers were excluded in two studies (Byun et al. 2020; Shomurodov and Mirkhusanova 2023), included in five studies (Bianchi et al. 2008; Chiapasco et al. 2004, 2007, 2013; Felice et al. 2008), while the remaining three studies (Felice et al. 2009; Morad and Khojasteh 2013; Roccuzzo et al. 2007) did not report information about smoking status (Table 1).

#### 3.4.2 Type of Defects

All studies aimed to correct vertical ridge deficiencies to support dental implant placement. The mean initial VBG required across the studies ranged from 4.2 to 6.6 mm (Table 2). Four studies focused specifically on defects in the posterior mandible (Bianchi et al. 2008; Chiapasco et al. 2007; Felice et al. 2008, 2009), one study included defects in the mandible without restricting them to a particular region (Morad and Khojasteh 2013), while the remaining five studies reported the inclusion of defects in any area of the mouth without specifying a particular location (Byun et al. 2020; Shomurodov and Mirkhusanova 2023; Chiapasco et al. 2004, 2013; Roccuzzo et al. 2007) (Table 1).

#### 3.4.3 Type of Interventions

The included studies evaluated various surgical techniques for vertical ridge augmentation, differing in graft material type, use of barrier membranes, and surgical approaches (Table 1). Onlay techniques were investigated in five studies (Chiapasco et al. 2007, 2013; Felice et al. 2009; Morad and Khojasteh 2013; Roccuzzo et al. 2007). Chiapasco et al. (2007) and Roccuzzo et al. (2007) used autogenous mandibular ramus bone, Chiapasco et al. (2013), in addition to madibular ramus, utilized autogenous bone harvested from the calvarium, and Felice et al. (2009) used autogenous bone harvested from the ilium. Morad and Khojasteh (2013) used two layers of intraoral autogenous bone blocks covered with a layer of particulate bovine bone.

The addition of barrier membranes to onlay grafts was evaluated in two studies (Chiapasco et al. 2013; Roccuzzo et al. 2007). Chiapasco et al. (2013) applied pericranium over cortical bone blocks, while Roccuzzo et al. (2007) used titanium mesh as a barrier over autogenous bone blocks.

Inlay techniques were assessed in three studies (Bianchi et al. 2008; Felice et al. 2008, 2009). Felice et al. (2008) compared the use of interpositional anorganic bovine blocks with the use of interpositional autologous blocks harvested from the iliac crest. Autogenous iliac interpositional grafts were also used in the two other studies (Bianchi et al. 2008; Felice et al. 2009).

GBR was examined in three studies (Byun et al. 2020; Shomurodov and Mirkhusanova 2023; Chiapasco et al. 2004), with variations in the type of membrane and graft materials used. Chiapasco et al. (2004) used e‐PTFE membranes with particulate autogenous bone harvested from the ramus and symphysis. Byun et al. (2020) employed d‐PTFE membranes with particulate xenogeneic bone. Shomurodov and Mirkhusanova (2023) did not report the type of membrane used but utilized a 1:1 mix of autogenous and xenogeneic bone.

Tissue expansion with tunneling was investigated in two studies (Byun et al. 2020; Shomurodov and Mirkhusanova 2023). Both studies used self‐inflating tissue expanders for a 4‐week expansion period. Byun et al. (2020) used particulate xenogeneic bone for grafting along with a collagen membrane as a barrier, while Shomurodov and Mirkhusanova (2023) grafted a 1:1 mix of autogenous and xenogeneic bone but did not report the type of barrier membrane used.

Distraction osteogenesis was evaluated in three studies (Bianchi et al. 2008; Chiapasco et al. 2004, 2007). The reported consolidation periods varied slightly, with Chiapasco et al. (2004), (2007) reporting a period of 2.5–3.5 months and Bianchi et al. (2008) reporting a 3–4 month period.

Cortical tenting was evaluated in one study: Morad and Khojasteh (2013). This study used screws to tent the cortical plate and filled the scaffold with a 50:50 mix of autogenous and xenogeneic particulate bone.

The included studies followed different staging durations between the initial surgery and re‐entry surgery. Four studies (Bianchi et al. 2008; Felice et al. 2008, 2009; Morad and Khojasteh 2013) had a ≤ 4‐month staging period, three studies (Chiapasco et al. 2007, 2013; Roccuzzo et al. 2007) followed a 4–7 month period, two studies (Byun et al. 2020; Shomurodov and Mirkhusanova 2023) had a 6‐month period, and one study (Chiapasco et al. 2004) had the longest staging period of 6–7 months.

#### 3.4.4 Measurement of Premature Bone Resorption (PBR)

PBR was measured using either radiographic or clinical (i.e., intrasurgical) methods across included studies. Radiographic assessments were conducted using computed tomography/cone beam computed tomography (CT/CBCT) in four studies (Byun et al. 2020; Shomurodov and Mirkhusanova 2023; Bianchi et al. 2008; Felice et al. 2009), periapical radiographs in three studies (Chiapasco et al. 2004, 2007; Felice et al. 2008), and panoramic radiographs (orthopantomograms, OPGs) in two studies (Chiapasco et al. 2013; Morad and Khojasteh 2013). Clinical measurements, using a periodontal probe, were performed in one study by Roccuzzo et al. (2007).

### 3.5 Results Synthesis

#### 3.5.1 Treatments' Grouping

The treatment groups in this analysis were categorized into seven categories: onlay, onlay + barrier, inlay, GBR, cortical tenting (CT), tissue expansion + tunneling (TET), and distraction osteogenesis (DO). These categories were formed after a careful evaluation of the clinical techniques, materials used, and biological principles underlying the interventions in the included studies. The grouping aimed to ensure consistency within each category, minimizing heterogeneity across studies. For example, studies involving the use of barrier membranes combined with onlay grafts were grouped separately from those using onlay grafts alone, reflecting the additional protective function of the membranes. Similarly, techniques partially preserving the periosteum (i.e., inlay and TET) were grouped separately from those requiring full flap elevation and from each other due to their unique surgical approaches and biological considerations.

#### 3.5.2 Effect of Interventions

##### 3.5.2.1 Premature Bone Resorption

**FIGURE 2:**
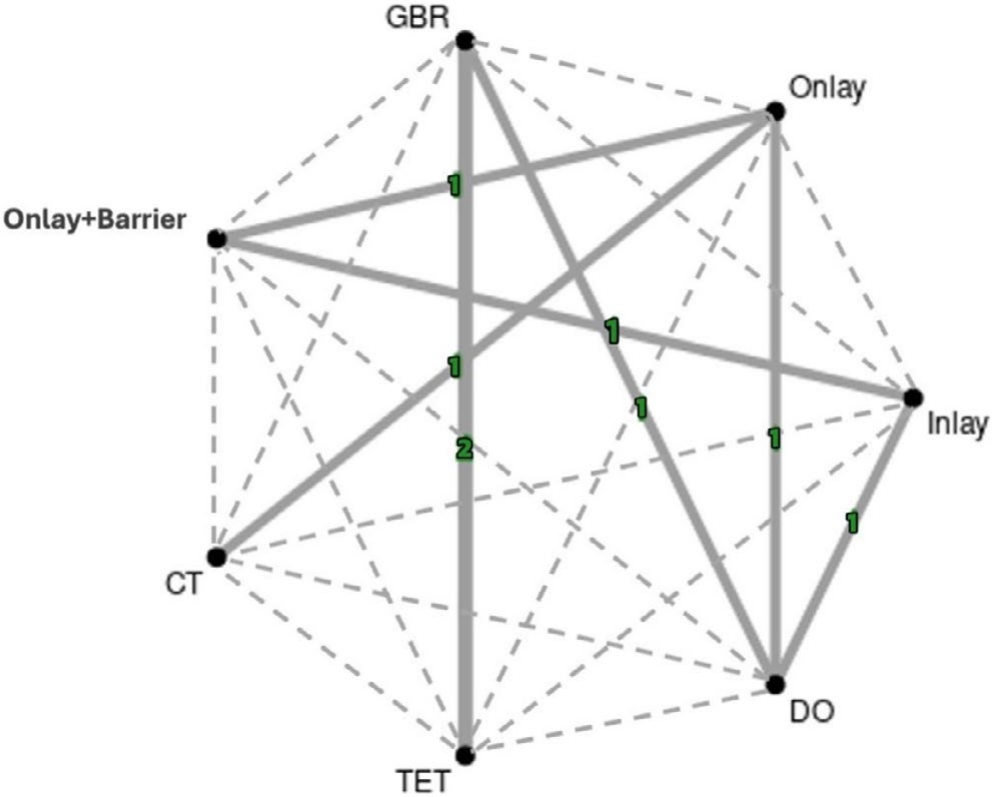
Network diagram for primary outcome: premature bone resorption. Solid lines refer to direct comparisons, while dotted lines refer to indirect comparisons. Numbers on solid lines refer to the number of studies included for each comparison.

Relative premature bone resorption was obtained for 9 of the 10 included studies (Table 2). Mean relative premature bone resorption for included interventions ranged from 6% to 44% with an average of 26%. Two of the 10 included studies did not contribute to the quantitative analysis; Chiapasco et al. (2013) did not report relative bone resorption and Felice et al. (2008) compared two interventions within the same NMA group. All treatment groups (*n* = 7) were analysed for relative premature bone resorption with a total of 21 possible comparisons (7 direct comparisons based on data from 8 RCTs, involving 160 participants and 176 defects, and 14 indirect comparisons) (Figure 2; Table 3).

**FIGURE 3:**
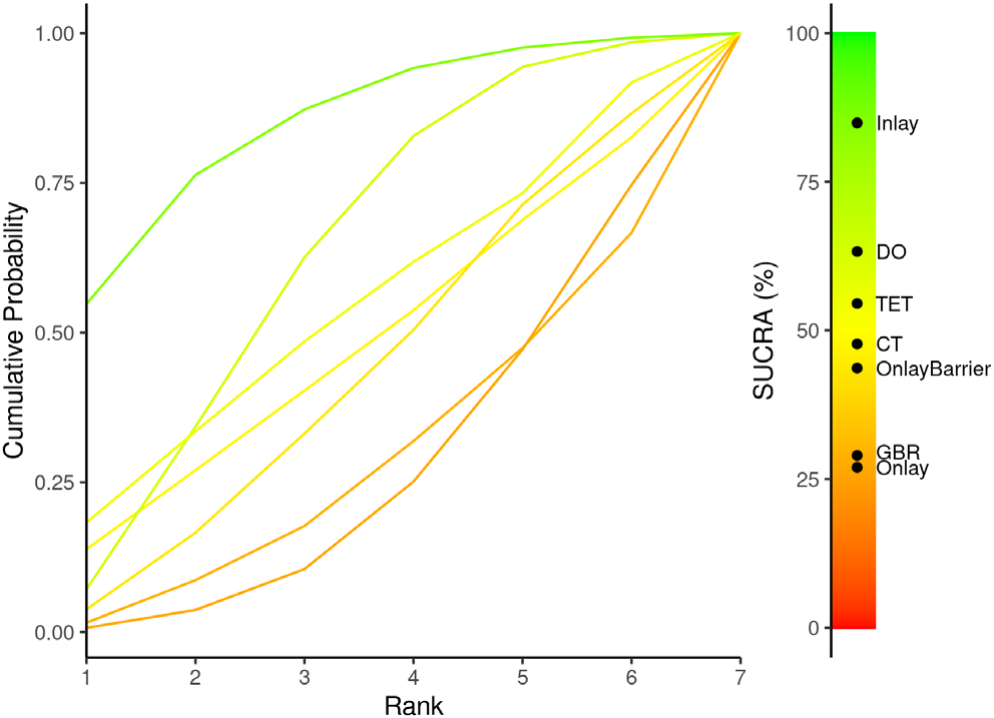
The surface under the cumulative ranking curve (SUCRA) for the primary outcome; premature bone resorption. Higher SUCRA values and cumulative ranking curves nearer the top left indicate better performance.

As the most connected group, Onlay served as a common comparator for the NMA analysis, which showed that, when compared to Onlay, all treatment groups had lower MD of relative premature bone resorption, with Inlay being the lowest (MD −26.5%, 95% CrI = −78.63, 23.7). None of the differences was statistically significant (Table 3). The treatment group that had the highest probability (Pr) to be ranked number one was the Inlay (Pr = 0.55, SUCRA = 84.89) followed by the DO (Pr = 0.27, SUCRA = 63.27) and then the TET (Pr = 0.15, SUCRA = 54.53) (Figure 3).

Insubstantial heterogeneity between studies was observed based on the inter‐studies SD 95% CrI (3.65, 28.97). The node‐splitting models included 4 observations with no statistically significant difference between direct and indirect comparisons (*p* > 0.05). The treatment group “Onlay” was identified as the most connected treatment, serving as the common comparator across the network. Transitivity was assessed by examining the distribution of patient characteristics, the similarity of defects and interventions, and the consistency in study design across comparisons. Only studies involving medically uncompromised adult patients with similar levels of oral hygiene, all having experienced tooth loss, and conducted as RCTs were included. All interventions were aimed at reconstructing vertical alveolar bone height for the purpose of placing dental implants and followed comparable surgical protocols. Publication bias was not assessed as the test for funnel plot asymmetry could not be performed with fewer than 10 studies contributing to the meta‐analysis.

##### 3.5.2.2 Premature Bone Resorption in Sites With Uneventful Healing Versus Sites With Healing Complications

**FIGURE 4:**
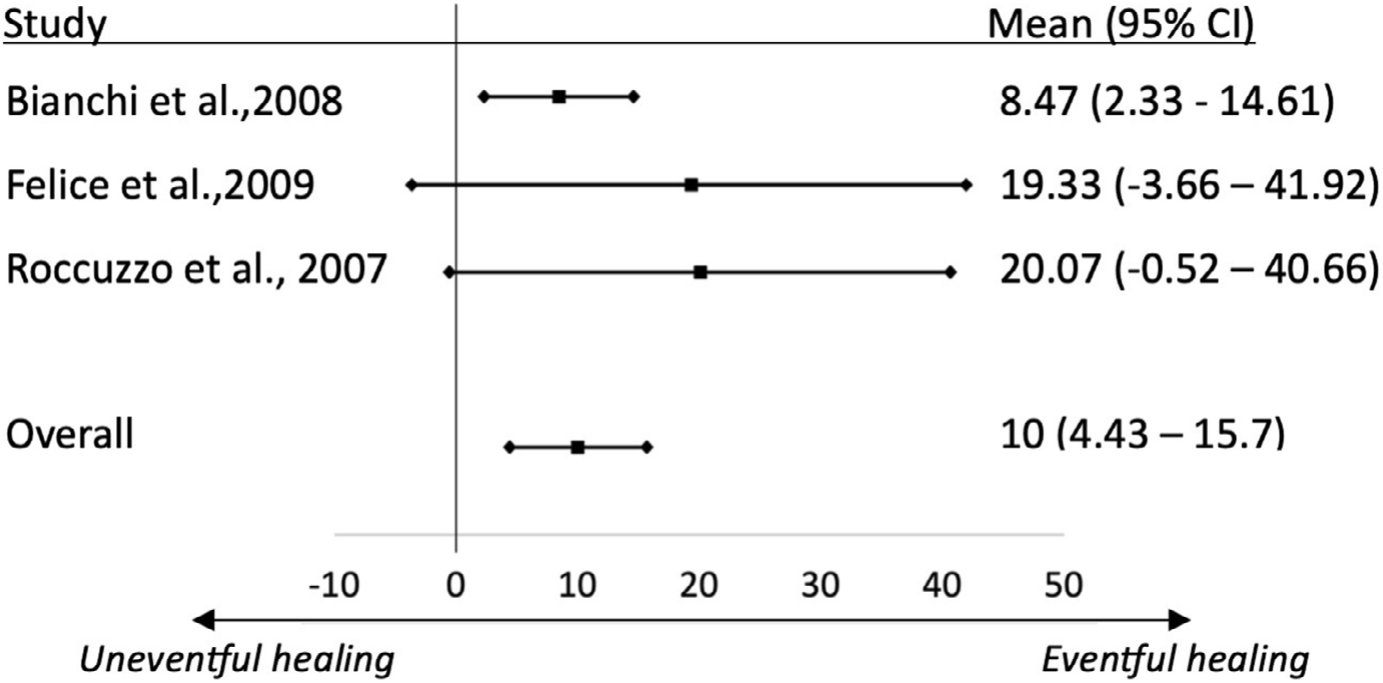
Regression analysis exploring the correlation between the incidence of healing complications and premature bone resorption.

Three RCTs reported this outcome (Table 1). Overall, 51 sites were included in the pair‐wise meta‐analysis, with 37 sites having uneventful healing and 14 experiencing healing complications. Healing complications reported in the studies included local infection, wound dehiscence, incomplete graft integration, and significant graft resorption. The analysis showed that sites with healing complications had significantly higher premature bone resorption, with an MD of 10% (95% CI = 4.43–15.7) (Figure 4).

##### 3.5.2.3 Need for Additional Grafting

Only one study reported this outcome. Roccuzzo et al. (2007) compared the use of autologous onlay blocks with or without titanium mesh coverage. Additional bone grafting was needed in 2 of the 12 cases in the coverage group (16.67%) and in 5 of the 12 cases in the no‐coverage group (41.67%).

## 4 Discussion

This systematic review and network meta‐analysis suggested that inlay (interpositional) blocks grafting results in the least amount of premature resorption, followed by distraction osteogenesis and then techniques that utilize soft tissue expansion prior to grafting. Healing complications were found to substantially increase the amount of premature bone resorption. Ten RCTs with a total population of 220 participants and 236 vertical ridge defects which were treated with either bone augmentation procedures or distraction osteogenesis had been reviewed. Onlay, being the most connected treatment in the network, was considered the common comparator for this analysis, facilitating indirect comparisons among the other interventions. The certainty of the evidence ranged from low to very low, owing to imprecision and concerns of risk of bias in the included studies. This is one of the few critical appraisals focused on the topic of premature bone resorption.

Inlay techniques resulted in 26.5% less premature resorption (95% CrI = −78.63, 23.7) when compared to onlay techniques. This approach involves making a paracrestal incision to access the facial side of the vertically deficient ridge, which is then split, raised, and stabilized, preserving the lingual periosteum attached to the cephalic (coronal) segment, thereby maintaining its blood supply (Felice et al. 2008, 2009). It could be hypothesized that the stabilization of the cephalic segment while keeping its blood supply partially intact, via lingual periosteum preservation, plays a role in reducing premature bone resorption. Nevertheless, the amount of bone gain that can be achieved in vertical dimension with this technique is constrained by the extent to which the lingually attached periosteum can be stretched, while no bone can be gained in horizontal dimensions. Additionally, the technique can only be applied when the initial defect has a minimum ridge height of at least 5 mm to allow for ridge splitting, which further limits its clinical applicability.

Pre‐augmentation soft tissue expansion resulted in 10% less premature graft resorption (95% CrI = −46.55, 68.95) compared to onlay, with a mean VBG of 3.5 mm (±1.56) and a 13% incidence of healing complications. All healing complications occurred during the tissue expansion period, which lasted 4 weeks in the included studies, and included tearing of the silicon envelope and perforation of the overlaying soft tissue. The concept of tissue expansion is based on the biological properties of various tissues, such as mucous membranes, to react to applied mechanical forces by true tissue growth resulting in a surplus amount of tissue which leads to reduced tension on wound edges and underlying grafts after surgical closure (Uijlenbroek et al. 2015). The technique's efficacy in vertical ridge augmentation has been reported with conflicting results in multiple reviews of preclinical and clinical studies. While initial results have been promising, these reviews concluded that the technique remains in a preliminary phase and requires further clinical investigations to establish clinical guidelines and protocols and to define its indications and contraindications (Asa'Ad et al. 2016; Medikeri et al. 2020).

When compared to onlay blocks alone, the use of barriers to cover bone blocks (i.e., onlay + barrier) or over particulate bone (i.e., GBR) resulted in 6.9% (95% CrI = −58.27, 63.71) and 0.23% (95% CrI = −50.75, 51.78) less premature resorption, respectively. In addition to their protective functions, membranes are reported to elicit favorable biological events linked to bone regeneration and remodeling (Omar et al. 2019). Non‐resorbable barriers, in particular, offer physical support that protects grafts from the tension of overlying soft tissues, which could exacerbate premature bone resorption.

Three RCTs reported individual patients' data, including the amount of vertical bone gain and incidence of complications. Our analysis of the reported data confirmed that healing complications lead to a significantly higher premature bone resorption (10%, 95% CI = 4.43, 15.7) when compared to cases that had an uneventful healing. This is in line with previous reviews that highlighted the significant impact of healing complications on the amount of bone gain (Tay et al. 2022).

Need for additional bone grafting at re‐entry was reported in a single RCT (Roccuzzo et al. 2007) and was required in approximately one‐third of the cases. Owing to the limited data, meaningful comparisons between the techniques included in this review were not possible. Nevertheless, previous reviews have reported a wide range of incidence of the need for additional grafting (Alotaibi et al. 2023; Naenni et al. 2019). Further studies are needed to fully investigate this question.

Limitations in this study should be highlighted and include the relatively limited number of available studies for various techniques and treatment groups, despite our broad inclusion criteria, necessitating cautious interpretation of the findings. The data did not permit a comprehensive evaluation of the impact of smoking and other patient‐related factors on successful or unsuccessful outcomes. Additionally, it is acknowledged that the difficulty of performing VRA varies significantly based on the location and three‐dimensional extent of the vertical defect, as well as the classification of edentulism (Kennedy I–IV). Unfortunately, due to constraints in the available data, sub‐analyses to investigate the effects of these factors were not feasible. Likewise, it is recognised that data from split‐mouth design studies require different statistical analysis methods, are prone to carryover effects, and may be less generalisable than data from parallel studies, ideally warranting separate consideration. However, due to the limited number of studies, sensitivity analyses based on the study types were not feasible, and both were evaluated using the same methodological approach. Readers are advised to interpret the results with caution in light of these limitations.

On the other hand, our systematic review was based on a pre‐registered protocol and adhered to a rigorous analytical approach, including all reported staged VRA techniques, potential confounding factors, and indirect comparisons. This comprehensive approach instills greater confidence in interpreting the results of the reported analyses.

## 5 Conclusion

There is a relatively small number of studies reporting PBR, limiting the certainty of the evidence. The findings of this review suggest that VRA techniques preserving the periosteum, including inlay, DO, and TET techniques, exhibit less premature bone resorption when compared with other techniques. They also suggest that with surgical techniques involving full flap elevation, clinicians should be aware of possible loss of volume at re‐entry and consider greater grafting volumes to compensate for PBR. Authors should aim to include data on premature bone resorption to inform future research and improve clinical evidence.

## Author Contributions

**Faisal Faihan Alotaibi:** conceptualization, investigation, funding acquisition, writing – original draft, writing – review and editing, visualization, validation, methodology, software, formal analysis, project administration, resources, data curation. **Jacopo Buti:** methodology, software, formal analysis, supervision, writing – review and editing. **Isabella Rocchietta:** methodology, validation, writing – review and editing, formal analysis, supervision. **Nor Shafina Mohamed Nazari:** data curation, investigation, validation, writing – review and editing, methodology. **Rawan Almujaydil:** validation, visualization, writing – review and editing, investigation. **Francesco D'Aiuto:** conceptualization, methodology, validation, investigation, writing – review and editing, project administration, supervision, resources, visualization.

## Disclosure

The authors have nothing to report.

## Ethics Statement

Ethics approval was not required for this systematic review. Nonetheless, all research steps were conducted in line with the principles of research ethics.

## Conflicts of Interest

The authors declare no conflicts of interest.

## Supporting information


Appendix S1.

## Data Availability

Data are available from the corresponding author upon reasonable request.
