# Supplementary material for: Premature Bone Resorption in Vertical Ridge Augmentation: A Systematic Review and Network Meta‐Analysis of Randomised Clinical Trials
Source: Clin Oral Implants Res. 2025 Mar 21;36(7):787–801. doi: 10.1111/clr.14435 (PMC12230887; doi:10.1111/clr.14435)
Supplement: Supplementary file 1 — Appendix S1. [file CLR-36-787-s001.docx]

**Appendix 1.1: Databases’ search strategies**

Database(s): Ovid MEDLINE(R) and Epub Ahead of Print, In-Process & Other Non-Indexed Citations and Daily 1946 to June 14, 2024
Search Strategy:

| # | Search | Hits |
| --- | --- | --- |
| 1 | exp Alveolar Ridge Augmentation/ | 4856 |
| 2 | (Alveolar Ridge adj3 Augmentation*).tw. | 633 |
| 3 | (Mandibular Ridge adj3 Augmentation*).tw. | 44 |
| 4 | (Maxillary Ridge adj3 Augmentation*).tw. | 21 |
| 5 | (alveolar bone adj3 augmentation*).tw. | 176 |
| 6 | (vertical ridge adj3 augmentation*).tw. | 201 |
| 7 | (vertical bone adj3 augmentation*).tw. | 227 |
| 8 | (alveolar bone adj3 graft*).tw. | 880 |
| 9 | (alveolar ridge adj3 graft*).tw. | 137 |
| 10 | exp Bone Regeneration/ | 29715 |
| 11 | bone regeneration.tw. | 18060 |
| 12 | exp Bone Transplantation/ | 34678 |
| 13 | bone transplantation.tw. | 731 |
| 14 | exp Osteogenesis, Distraction/ | 5040 |
| 15 | distraction osteogenesis.tw. | 4394 |
| 16 | exp Allografts/ | 12314 |
| 17 | allograft*.tw. | 77168 |
| 18 | allogenic graft*.tw. | 158 |
| 19 | allogenic transplant*.tw. | 482 |
| 20 | allotransplant*.tw. | 5326 |
| 21 | exp Autografts/ | 4288 |
| 22 | autograft*.tw. | 20254 |
| 23 | (autologous adj3 transplant*).tw. | 21045 |
| 24 | (autologous adj3 graft*).tw. | 10003 |
| 25 | autotransplant*.tw. | 7634 |
| 26 | exp Heterografts/ | 10769 |
| 27 | heterograft*.tw. | 1027 |
| 28 | heterotransplant*.tw. | 1078 |
| 29 | xenograft*.tw. | 91863 |
| 30 | xenotransplant*.tw. | 8720 |
| 31 | exp Bone Substitutes/ | 10494 |
| 32 | (alloplastic adj3 graft*).tw. | 375 |
| 33 | (alloplastic adj3 transplant*).tw. | 34 |
| 34 | interpositional graft*.tw. | 277 |
| 35 | (bone adj3 block).tw. | 1948 |
| 36 | tent pole.tw. | 32 |
| 37 | tenting.tw. | 763 |
| 38 | exp Polytetrafluoroethylene/ | 11815 |
| 39 | Polytetrafluoroethylene.tw. | 9222 |
| 40 | PTFE.tw. | 6749 |
| 41 | exp Titanium/ | 47972 |
| 42 | titanium mesh.tw. | 1725 |
| 43 | exp Surgical Mesh/ | 17363 |
| 44 | surgical mesh.tw. | 414 |
| 45 | exp Guided Tissue Regeneration, Periodontal/ | 3529 |
| 46 | (guided adj3 regeneration).tw. | 4493 |
| 47 | "short implant*".tw. | 740 |
| 48 | or/1-47 | 389525 |
| 49 | exp Bone Resorption/ and (oral or mouth or dental).tw. | 6267 |
| 50 | ((bone adj3 resorp*) and (oral or implant* or mouth or dental)).tw. | 6519 |
| 51 | ((bone adj3 deficien*) and (oral or implant* or mouth or dental)).tw. | 733 |
| 52 | (ridge adj3 resorp*).tw. | 473 |
| 53 | (ridge adj3 deficien*).tw. | 247 |
| 54 | ((bone adj3 defect*) and (oral or mouth or dental)).tw. | 1935 |
| 55 | (ridge adj3 defect*).tw. | 373 |
| 56 | exp Mouth, Edentulous/ | 13025 |
| 57 | Edentulous.tw. | 13608 |
| 58 | exp Dental Implants/ | 30745 |
| 59 | dental implant*.tw. | 20100 |
| 60 | exp Dental Implantation/ | 24447 |
| 61 | exp Mouth Rehabilitation/ | 1484 |
| 62 | mouth rehabilitation.tw. | 419 |
| 63 | dent* rehabilitation.tw. | 833 |
| 64 | *dental prosthesis/ or exp dental implants/ or exp dental prosthesis, implant-supported/ | 37112 |
| 65 | dental prosthes?s.tw. | 4204 |
| 66 | implant-supported.tw. | 6626 |
| 67 | or/49-66 | 78243 |
| 68 | randomi?ed controlled trial.pt. | 615435 |
| 69 | controlled clinical trial.pt. | 95556 |
| 70 | randomized.ab. | 649281 |
| 71 | placebo.ab. | 249175 |
| 72 | drug therapy.fs. | 2705130 |
| 73 | randomly.ab. | 435597 |
| 74 | trial.ab. | 702102 |
| 75 | groups.ab. | 2691906 |
| 76 | or/68-75 | 5986293 |
| 77 | exp animals/ not humans.sh. | 5231654 |
| 78 | 76 not 77 | 5237647 |
| 79 | 48 and 67 and 78 | 3921 |

Database(s): Embase <1974 to 2022 June 14, 2024>
Search Strategy:

| # | Search | Hits |
| --- | --- | --- |
| 1 | exp alveolar ridge augmentation/ | 1328 |
| 2 | (Alveolar Ridge adj3 Augmentation*).tw. | 623 |
| 3 | (Mandibular Ridge adj3 Augmentation*).tw. | 43 |
| 4 | (Maxillary Ridge adj3 Augmentation*).tw. | 22 |
| 5 | (alveolar bone adj3 augmentation*).tw. | 173 |
| 6 | (vertical ridge adj3 augmentation*).tw. | 208 |
| 7 | (vertical bone adj3 augmentation*).tw. | 223 |
| 8 | (alveolar bone adj3 graft*).tw. | 1005 |
| 9 | (alveolar ridge adj3 graft*).tw. | 130 |
| 10 | exp bone regeneration/ | 40119 |
| 11 | bone regeneration.tw. | 19255 |
| 12 | *bone transplantation/ or *bone graft/ | 18413 |
| 13 | bone transplantation.tw. | 797 |
| 14 | *distraction osteogenesis/ | 3945 |
| 15 | distraction osteogenesis.tw. | 5034 |
| 16 | *allograft/ or *transplantation/ or *bone allograft/ | 77175 |
| 17 | allograft*.tw. | 113433 |
| 18 | allogenic graft*.tw. | 201 |
| 19 | allogenic transplant*.tw. | 984 |
| 20 | allotransplant*.tw. | 6980 |
| 21 | *autograft/ or *transplantation/ | 68758 |
| 22 | autograft*.tw. | 24451 |
| 23 | (autologous adj3 transplant*).tw. | 39105 |
| 24 | (autologous adj3 graft*).tw. | 12124 |
| 25 | autotransplant*.tw. | 9221 |
| 26 | exp xenograft/ | 53064 |
| 27 | heterograft*.tw. | 816 |
| 28 | heterotransplant*.tw. | 882 |
| 29 | xenograft*.tw. | 139799 |
| 30 | xenotransplant*.tw. | 12644 |
| 31 | *bone prosthesis/ | 5012 |
| 32 | (alloplastic adj3 graft*).tw. | 404 |
| 33 | (alloplastic adj3 transplant*).tw. | 31 |
| 34 | interpositional graft*.tw. | 322 |
| 35 | *bone graft/ or *bone allograft/ or *synthetic bone graft/ | 13211 |
| 36 | (bone adj3 block).tw. | 2230 |
| 37 | tent pole.tw. | 41 |
| 38 | tenting.tw. | 1401 |
| 39 | *politef/ | 6452 |
| 40 | Polytetrafluoroethylene.tw. | 10313 |
| 41 | PTFE.tw. | 8827 |
| 42 | exp titanium mesh/ or exp surgical mesh/ | 25984 |
| 43 | titanium mesh.tw. | 2029 |
| 44 | surgical mesh.tw. | 618 |
| 45 | tissue regeneration/ or *periodontal guided tissue regeneration/ | 35763 |
| 46 | (guided adj3 regeneration).tw. | 4472 |
| 47 | "short implants*".tw. | 615 |
| 48 | or/1-47 | 541527 |
| 49 | exp osteolysis/ and (oral or mouth or dental).tw. | 8815 |
| 50 | ((bone adj3 resorp*) and (oral or implant* or mouth or dental)).tw. | 7795 |
| 51 | ((bone adj3 deficien*) and (oral or implant* or mouth or dental)).tw. | 888 |
| 52 | *bone defect/ and (oral or implant* or mouth or dental).tw. | 2489 |
| 53 | (ridge adj3 resorp*).tw. | 478 |
| 54 | (ridge adj3 deficien*).tw. | 234 |
| 55 | ((bone adj3 defect*) and (oral or mouth or dental)).tw. | 2384 |
| 56 | (ridge adj3 defect*).tw. | 404 |
| 57 | exp edentulousness/ | 1047 |
| 58 | Edentulous*.tw. | 13547 |
| 59 | exp tooth implant/ | 22639 |
| 60 | dental implant*.tw. | 20048 |
| 61 | tooth implant*.tw. | 1027 |
| 62 | exp tooth implantation/ | 28909 |
| 63 | exp full mouth rehabilitation/ | 467 |
| 64 | mouth rehabilitation.tw. | 342 |
| 65 | dent* rehabilitation.tw. | 933 |
| 66 | exp tooth prosthesis/ or exp "dental prosthesis and implant"/ or exp prosthesis/ or exp tooth implant/ | 247841 |
| 67 | dental prosthes?s.tw. | 3882 |
| 68 | exp implant-supported denture/ | 2568 |
| 69 | implant-supported.tw. | 6263 |
| 70 | or/49-69 | 281914 |
| 71 | 48 and 70 | 25377 |
| 72 | randomized controlled trial/ | 826901 |
| 73 | controlled clinical study/ | 473400 |
| 74 | random*.tw. | 2080447 |
| 75 | randomisation/ | 99453 |
| 76 | intermethod comparison/ | 307084 |
| 77 | placebo.tw. | 378553 |
| 78 | (compare or compared or comparison).ti. | 627422 |
| 79 | ((evaluated or evaluate or evaluating or assessed or assess) and (compare or compared or company or comparison)).ab. | 2836139 |
| 80 | (open adj label).tw. | 116129 |
| 81 | ((double or single or doubly or singly) adj (blind or blinded or blindly)).tw. | 283492 |
| 82 | double blind procedure/ | 220135 |
| 83 | parallel group*.tw. | 33780 |
| 84 | (crossover or cross over).tw. | 128770 |
| 85 | ((assign* or match or matched or allocation) adj5 (alternate or group*1 or intervention*1 or patient*1 or subject*1 or participant*1)).tw. | 435338 |
| 86 | (assigned or allocated).tw. | 514504 |
| 87 | (controlled adj7 (study or design or trial)).tw. | 474374 |
| 88 | trial.ti. | 426782 |
| 89 | or/72-88 | 5917600 |
| 90 | ((exp animal/ or animal.hw. or nonhuman/) not (exp human/ or human cell/)) or (human or humans).ti. | 8681261 |
| 91 | 89 not 90 | 4994022 |
| 92 | 71 and 91 | 3748 |

Database: Web of Science Core Collection, June 16, 2024

Search Strategy:

| # | Search | Hits |
| --- | --- | --- |
| 1 | TS=(Ridge near/4 Augmentation*) OR TS=(alveolar bone near/3 augmentation*) OR TS=(alveolar bone near/3 graft*) OR TS=(ridge near/4 graft*) OR TS=("distraction osteogenesis") OR TS=(allograft*) OR TS=("allogenic graft*") OR TS=("allogenic transplant*") OR TS=(allotransplant*) OR TS=(autograft*) OR TS=(autologous near/3 transplant*) OR TS=(autologous near/3 graft*) OR TS=(autotransplant*) OR TS=(xenograft*) OR TS=(xenotransplant*) OR TS=("Bone Substitute*") OR TS=(heterograft*) OR TS=(heterotransplant*) OR TS=(alloplastic near/3 graft*) OR TS=(alloplastic near/3 transplant*) OR TS=("interpositional graft*") OR TS=(bone near/3 block) OR TS=("tent pole") OR TS=(tenting) OR TS=(Polytetrafluoroethylene) | 343832 |
| 2 | TS=(PTFE) OR TS=("titanium mesh") OR TS=("surgical mesh") OR TS=(guided near/3 regeneration*) | 31663 |
| 3 | TS=("alveolar bone resorp*") OR TS=("alveolar bone deficien*") OR TS=(ridge near/3 resorp*) OR TS=(ridge near/3 deficien*) OR TS=("alveolar bone defect*") OR TS=(ridge near3 defect*) OR TS=(Edentulous) OR TS=(dental implant) OR TS=(Mouth Rehabilitation*) OR TS=(dent* rehabilitation) OR TS=(dental prosthes?s) OR TS=(implant-supported) | 59864 |
| 4 | TS=(randomi?ed) OR TS=(trial) OR TS=(randomly) OR TS=(groups) | 8946046 |
| 5 | TS=(human) NOT TS=(animal or dog or mice or beagle*) | 4400625 |
| 6 | #1 OR #2 | 361910 |
| 7 | #6 AND #3 | 5630 |
| 8 | #7 AND #4 | 2185 |
| 9 | #8 AND #5 | 308 |

Database: Central, July 01, 2023

Search Strategy:

| # | Search | Hits |
| --- | --- | --- |
| 1 | MeSH descriptor: [Alveolar Ridge Augmentation] explode all trees | 492 |
| 2 | Alveolar ridge augmentation | 720 |
| 3 | MeSH descriptor: [Osteogenesis, Distraction] explode all trees | 98 |
| 4 | Distraction Osteogenesis | 179 |
| 5 | MeSH descriptor: [Autografts] explode all trees | 248 |
| 6 | Autograft* | 2108 |
| 7 | MeSH descriptor: [Allografts] explode all trees | 313 |
| 8 | Allograft* | 5246 |
| 9 | MeSH descriptor: [Transplantation, Autologous] explode all trees | 2005 |
| 10 | Autologous Transplant* | 7899 |
| 11 | MeSH descriptor: [Transplantation, Homologous] explode all trees | 1522 |
| 12 | Homologous Transplant* | 1571 |
| 13 | MeSH descriptor: [Heterografts] explode all trees | 108 |
| 14 | Heterograft* | 120 |
| 15 | MeSH descriptor: [Transplantation, Heterologous] explode all trees | 104 |
| 16 | Heterologous Transplant* | 144 |
| 17 | MeSH descriptor: [Bone Substitutes] 1 tree(s) exploded | 649 |
| 18 | Bone Substitute* | 1436 |
| 19 | MeSH descriptor: [Bone Transplantation] explode all trees | 1278 |
| 20 | Bone Transplant* | 9919 |
| 21 | interpositional graft* | 56 |
| 22 | alloplastic graft* | 85 |
| 23 | bone block | 1986 |
| 24 | MeSH descriptor: [Polytetrafluoroethylene] 1 tree(s) exploded | 514 |
| 25 | (PTFE) or (Polytetrafluoroethylene) | 1109 |
| 26 | MeSH descriptor: [Surgical Mesh] explode all trees | 1075 |
| 27 | Surgical Mesh | 4879 |
| 28 | MeSH descriptor: [Titanium] explode all trees | 1056 |
| 29 | titanium | 2961 |
| 30 | MeSH descriptor: [Guided Tissue Regeneration] explode all trees | 0 |
| 31 | Guided Tissue Regeneration | 1018 |
| 32 | MeSH descriptor: [Bone Regeneration] explode all trees | 1039 |
| 33 | (alloplastic adj3 transplant*).tw. | 10573 |
| 34 | ridge augment* | 862 |
| 35 | bone augment* | 3027 |
| 36 | bone graft* | 6597 |
| 37 | ridge graft* | 841 |
| 38 | #1 or #2 or #3 or #4 or #5 or #6 or #7 or #8 or #9 or #10 or #11 or #12 or #13 or #14 or #15 or #16 or #17 or #18 or #19 or #20 or #21 or #22 or #23 or #24 or #25 or #26 or #27 or #28 or #29 or #30 or #31 or #32 or #33 or #34 or #35 #36 or #37 | 41687 |
| 39 | MeSH descriptor: [Bone Resorption] explode all trees | 3110 |
| 40 | Bone Resorpt* | 4454 |
| 41 | MeSH descriptor: [Alveolar Bone Loss] explode all trees | 1546 |
| 42 | Alveolar Bone Loss | 2250 |
| 43 | Ridge Resorp* | 339 |
| 44 | bone deficien* | 3462 |
| 45 | ridge defect* | 294 |
| 46 | bone defect* | 3523 |
| 47 | MeSH descriptor: [Jaw, Edentulous] explode all trees | 690 |
| 48 | MeSH descriptor: [Mouth, Edentulous] explode all trees | 935 |
| 49 | Edentulous | 2368 |
| 50 | MeSH descriptor: [Dental Implants] explode all trees | 2065 |
| 51 | Dental Implant* | 5538 |
| 52 | MeSH descriptor: [Mouth Rehabilitation] explode all trees | 12 |
| 53 | dent* rehabilitation | 2113 |
| 54 | MeSH descriptor: [Dental Prosthesis] explode all trees | 5600 |
| 55 | Dental Prosthesis, Implant-Supported | 1096 |
| 56 | dental prosthes?s | 2730 |
| 57 | implant-supported | 1470 |
| #58 | #39 or #40 or #41 or #42 or #43 or #44 or #45 or #46 or #47 or #48 or #49 or #50 or #51 or #52 or #53 or #54 or #55 or #56 or #57 | 20993 |
| 59 | #38 and #58 | 5636 |
| 60 | (#59) in Trials with 'Oral Health' in Cochrane Groups | 757 (187 trials) |

Database: LILACS, June 14, 2024

Search strategy:

((alveolar ridge augmentation) OR (dental implant) OR (distraction osteogenesis)) AND ((alveolar ridge deficiency) OR (alveolar bone loss))

Database: Clinical trials.gov, June 14,2024

Search strategy:

“alveolar ridge deficiency” AND “alveolar ridge augmentation:

“alveolar bone loss” AND “alveolar ridge augmentation”

“alveolar ridge deficiency” AND “dental implants“

“alveolar bone loss” AND “dental implants”

“alveolar ridge deficiency” AND “distraction osteogenesis”

“alveolar bone loss” AND “distraction osteogenesis

**Appendix 1.2: Heterogeneity, inconsistency and transitivity**

**Heterogeneity**: prediction intervals were used to assess heterogeneity (Riley et al. 2011; Rücker et al. 2008). If the between-trials standard deviation [SD] and 95% CrI do not contain the no-effect value, this will indicate insubstantial heterogeneity between studies. If the no-effect value is contained, however, this will indicate substantial heterogeneity between studies.

**Inconsistency**: node-splitting models generated by Metainsight tool were used to assess inconsistency. The models calculate the statistical significance (p-value) of the difference between direct and indirect comparisons (Buti et al. 2011).

**Transitivity**: assessed by exploring the distribution of patient characteristics, similarity of interventions, and study design across comparisons. Based on the pre-determined set of inclusion and exclusion criteria, only adult patients who are medically uncompromised, with similar level of oral hygiene (have had tooth loss) and enrolled in a randomised clinical trial were included. Therefore, deviations in patient characteristics are more likely to be due chance, rather than real clinical difference.

**Appendix 1.3: Excluded papers with reasons for exclusion**

|  | Author(s) | Title | Journal | Volume | Issue | Year | Reason for execlusion |
| --- | --- | --- | --- | --- | --- | --- | --- |
|  | Abrahamsson, P.: Walivaara, D. A.: Isaksson, S.: Andersson, G. | Periosteal expansion before local bone reconstruction using a new technique for measuring soft tissue profile stability: a clinical study | Journal of Oral & Maxillofacial Surgery | 70 | 10 | 2012 | Horizontal ridge augmentation |
|  | Agarwal, A.: Gupta, N. D. | Alveolar ridge augmentation by connective tissue grafting using a pouch method and modified connective tissue technique: A prospective study | Dental Research Journal | 12 | 6 | 2015 | no bone graft |
|  | Akhlaghi, F.: Hesami, N.: Rad, M. R.: Nazeman, P.: Fahimipour, F.: Khojasteh, A. | Improved bone regeneration through amniotic membrane loaded with buccal fat pad-derived MSCs as an adjuvant in maxillomandibular reconstruction | Journal of Cranio-Maxillo-Facial Surgery | 47 | 8 | 2019 | non-randomised |
|  | Al-Kassaby, A.: Shindy, I.: Asker, N.: Ibrahim, M. | Evaluating the effect of low intensity pulsed ultrasound on the quality of regenerated bone in vertical alveolar osteodistraction | International Journal of Oral and Maxillofacial Surgery | 46 (Supplement 1) |  | 2017 | Only abstract available |
|  | Al-Kassaby, A.: Shindy, M. | Comparing the duration of different phases of vertical alveolar distraction between ultrasound treated and control group in anterior mandible | International Journal of Oral and Maxillofacial Surgery | 48 (Supplement 1) |  | 2019 | Only abstract available |
|  | Antoun, H.: Sitbon, J. M.: Martinez, H.: Missika, P. | A prospective randomized study comparing two techniques of bone augmentation: onlay graft alone or associated with a membrane | Clinical Oral Implants Research | 12 | 6 | 2001 | Horizontal ridge augmentation |
|  | Bettega, G.: Brun, J. P.: Cracowski, J. L.: Verain, A.: Raphael, B. | [Use of autologous platelet concentrates during pre-implantation maxillary reconstruction] | Revue de Stomatologie et de Chirurgie Maxillo-Faciale | 106 | 3 | 2005 | sinus left cases |
|  | Cansiz, E.: Haq, J.: Manisali, M.: Cakarer, S.: Gultekin, B. A. | Long-term evaluation of three-dimensional volumetric changes of augmented severely atrophic maxilla by anterior iliac crest bone grafting | Journal of stomatology, oral and maxillofacial surgery | 121 | 6 | 2020 | retrospective |
|  | Canullo, L.: Sisti, A. | Early implant loading after vertical ridge augmentation (VRA) using e-PTFE titanium-reinforced membrane and nano-structured hydroxyapatite: 2-year prospective study | European journal of oral implantology | 3 | 1 | 2010 | no controls |
|  | Castagna, L.: Polido, W. D.: Soares, L. G.: Tinoco, E. M. | Tomographic evaluation of iliac crest bone grafting and the use of immediate temporary implants to the atrophic maxilla | International Journal of Oral & Maxillofacial Surgery | 42 | 9 | 2013 | not comparing VRA techniques |
|  | Castry, G.: Ella, B.: Emparanza, A.: Siberchicot, F.: Zwetyenga, N. | Psychological impact of alveolar mandibular distraction. [French] | Revue de Stomatologie et de Chirurgie Maxillo-Faciale | 110 | 5 | 2009 | no controls |
|  | Chen, S. T.: Darby, I. B.: Adams, G. G.: Reynolds, E. C. | A prospective clinical study of bone augmentation techniques at immediate implants | Clinical Oral Implants Research | 16 | 2 | 2005 | subjects not diagnosed w\ ridge deficincey |
|  | Chiapasco, M.: Di Martino, G.: Anello, T.: Zaniboni, M.: Romeo, E. | Fresh frozen versus autogenous iliac bone for the rehabilitation of the extremely atrophic maxilla with onlay grafts and endosseous implants: preliminary results of a prospective comparative study | Clinical Implant Dentistry & Related Research | 17 Suppl 1 |  | 2015 | no randomization |
|  | Cucchi, A.: Vignudelli, E.: Fiorino, A.: Pellegrino, G.: Corinaldesi, G. | Vertical ridge augmentation (VRA) with Ti-reinforced d-PTFE membranes or Ti-meshes and collagen membranes. 1-year results of a randomized clinical trial | Clinical oral implants research. | 5 |  | 2020 | dublicate |
|  | D'Amato, S.: Tartaro, G.: Itro, A.: Nastri, L.: Santagata, M. | Block versus particulate/titanium mesh for ridge augmentation for mandibular lateral incisor defects: clinical and histologic analysis | International Journal of Periodontics & Restorative Dentistry | 35 | 1 | 2015 | Horizontal Defects |
|  | Dasmah, A.: Thor, A.: Ekestubbe, A.: Sennerby, L.: Rasmusson, L. | Particulate vs. block bone grafts: Three-dimensional changes in graft volume after reconstruction of the atrophic maxilla, a 2-year radiographic follow-up | Journal of Cranio-Maxillofacial Surgery | 40 | 8 | 2012 | sinus grafts |
|  | Dasmah, A.: Thor, A.: Ekestubbe, A.: Sennerby, L.: Rasmusson, L. | Marginal bone-level alterations at implants installed in block versus particulate onlay bone grafts mixed with platelet-rich plasma in atrophic maxilla. a prospective 5-year follow-up study of 15 patients | Clinical Implant Dentistry & Related Research | 15 | 1 | 2013 | non-randomised |
|  | Dellavia, C.: Giammattei, M.: Carmagnola, D.: Musto, F.: Canciani, E.: Chiapasco, M. | Iliac Crest Fresh-Frozen Allografts Versus Autografts in Oral Pre-Prosthetic Bone Reconstructive Surgery: Histologic and Histomorphometric Study | Implant Dentistry | 25 | 6 | 2016 | non-randomised |
|  | Deluiz, D.: Oliveira, L. S.: Pires, F. R.: Tinoco, E. M. | Time-dependent changes in fresh-frozen bone block grafts: tomographic, histologic, and histomorphometric findings | Clinical Implant Dentistry & Related Research | 17 | 2 | 2015 | compares healing periods |
|  | Dursun, E.: Keceli, H. G.: Uysal, S.: Gungor, H.: Muhtarogullari, M.: Tozum, T. F. | Management of Limited Vertical Bone Height in the Posterior Mandible: Short Dental Implants Versus Nerve Lateralization With Standard Length Implants | Journal of Craniofacial Surgery | 27 | 3 | 2016 | no outcomes of interest |
|  | Eisenbraun, O.: Tarasenko, S. | Achieving enhanced bone augmentation via tunnel technique | International Journal of Oral and Maxillofacial Surgery | 44 |  | 2015 | Only abstract available |
|  | El Hadidy, M. S.: Mounir, M.: Abou-Elfetouh, A.: Barakat, A. | Assessment of vertical ridge augmentation and labial prominence using buccal versus palatal approaches for maxillary segmental sandwich osteotomy (inlay technique): A randomized clinical trial | Clinical Implant Dentistry & Related Research | 20 | 5 | 2018 | No outcome of interst |
|  | Yuan, S.: Chen, T.: Li, D. Z.: Huang, Y. D.: Tang, H.: Ji, P. | [Assessment of the clinical effect regarding three-dimensional printing individualized titanium mesh for bone augmentation in the esthetic area] | Chung-Hua Kou Chiang i Hsueh Tsa Chih Chinese Journal of Stomatology | 55 | 11 | 2020 | not RCT |
|  | Felice, P.: Marchetti, C.: Piattelli, A.: Pellegrino, G.: Checchi, V.: Worthington, H.: Esposito, M. | Vertical ridge augmentation of the atrophic posterior mandible with interpositional block grafts: bone from the iliac crest versus bovine anorganic bone | European Journal of Oral Implantology | 1 | 3 | 2008 | dublicate |
|  | Garcia-Garcia, A.: Somoza-Martin, M. | Distraction osteogenesis with subperiosteal devices in edentulous mandibles | British Journal of Oral and Maxillofacial Surgery | 45 | 2 | 2007 | letter for editors |
|  | Hassan, S.: Sadek, H.: Tantawi, E. | Bone graft remodeling after ridge reconstruction with autogenous bone and statin | International Journal of Oral and Maxillofacial Surgery | 40 (10) |  | 2011 | Only abstract available |
|  | Ugurlu, F.: Sener, B. C.: Sertgoz, A. | The effects of different consolidation periods on bone formation and implant success in alveolar distraction osteogenesis: A clinical study | International Journal of Oral and Maxillofacial Surgery | 40 (10) |  | 2011 | Only abstract available |
|  | Lizio, G.: Felice, P.: Pellegrino, G.: Pistilli, R.: Marchetti, C. | Ridge augmentation of the atrophic posterior mandible with inlay and onlay iliac bone grafting : A prospective controlled clinical trial for the comparison of two techniques | European Surgical Research | 45 (3-4) |  | 2010 | poster only |
|  | Alessandro Cucchi | Reinforced PTFE Meshes Versus Customized Titanium Meshes | <https://clinicaltrials.gov/show/NCT04257097> |  |  | 2020 | protocol |
|  | Schlegel, K. A.: Karascholi, T.: Fenner, M.: Nkenke, E. |  | Mund - Kiefer - und Gesichtschirurgie | 11 | 4 | 2007 | not RCT |
|  | Felice, P.:Soardi, E.:Pellegrino, G.:Pistilli, R.:Marchetti, C.:Gessaroli, M.:Esposito, M. | Treatment of the atrophic edentulous maxilla: short implants versus bone augmentation for placing longer implants. Five-month post-loading results of a pilot randomised controlled trial | European Journal of Oral Implantology | 4 | 3 | 2011 | Sinus floor elevation |
|  | Hadzik, J.:Krawiec, M.:Kubasiewicz-Ross, P.:Prylinska-Czyzewska, A.:Gedrange, T.:Dominiak, M. | Short Implants and Conventional Implants in The Residual Maxillary Alveolar Ridge: A 36-Month Follow-Up Observation | Medical Science Monitor | 24 |  | 2018 | Sinus floor elevation |
|  | Kaigler, D.:Pagni, G.:Park, C. H.:Braun, T. M.:Holman, L. A.:Yi, E.:Tarle, S. A.:Bartel, R. L.:Giannobile, W. V. | Stem cell therapy for craniofacial bone regeneration: a randomized, controlled feasibility trial | Cell Transplantation | 22 | 5 | 2013 | test isn’t VRA or SI |
|  | Khojasteh, A.:Sadeghi, N. | Application of buccal fat pad-derived stem cells in combination with autogenous iliac bone graft in the treatment of maxillomandibular atrophy: a preliminary human study | International Journal of Oral and Maxillofacial Surgery | 45 | 7 | 2016 | non-randomized |
|  | Kim, J. W.:Cho, M. H.:Kim, S. J.:Kim, M. R. | Alveolar distraction osteogenesis versus autogenous onlay bone graft for vertical augmentation of severely atrophied alveolar ridges after 12 years of long-term follow-up | Oral Surgery, Oral Medicine, Oral Pathology and Oral Radiology | 116 | 5 | 2013 | retrospective |
|  | Eskan, M. A.:Greenwell, H.:Hill, M.:Morton, D.:Vidal, R.:Shumway, B.:Girouard, M. E. | Platelet-rich plasma-assisted guided bone regeneration for ridge augmentation: a randomized, controlled clinical trial | Journal of Periodontology | 85 | 5 | 2014 | Horizontal ridge augmentation and less than 6 mnths f\u |
|  | Maiorana, C.:Beretta, M.:Salina, S.:Santoro, F. | Reduction of autogenous bone graft resorption by means of bio-oss coverage: a prospective study | International Journal of Periodontics & Restorative Dentistry | 25 | 1 | 2005 | Horizontal ridge augmentation |
|  | Mazzonetto, R.:Serra, E. Silva F. M.:Ribeiro Torezan, J. F. | Clinical assessment of 40 patients subjected to alveolar distraction osteogenesis | Implant Dentistry | 14 | 2 | 2005 | no comparsion |
|  | Menezes, D. J. B.:Shibli, J. A.:Gehrke, S. A.:Beder, A. M.:Sendyk, W. R. | Effect of platelet-rich plasma in alveolar distraction osteogenesis: A controlled clinical trial | British Journal of Oral and Maxillofacial Surgery | 54 | 1 | 2016 | No outcome of interest |
|  | Zitzmann, N. U.:Naef, R.:Scharer, P. | Resorbable versus nonresorbable membranes in combination with Bio-Oss for guided bone regeneration | International Journal of Oral & Maxillofacial Implants | 12 | 6 | 1997 | cases of exposed imp surface |
|  | Hazzaa, H. H. A.:El-Kilani, N. S.:Elsayed, S. A.:Abd El Massieh, P. M. | Evaluation of Immediate Implants Augmented with Autogenous Bone/Melatonin Composite Graft in the Esthetic Zone: A Randomized Controlled Trial | Journal of Prosthodontics | 28 | 2 | 2019 | socket preservation |
|  | Nogami, S.:Yamauchi, K.:Shiiba, S.:Kataoka, Y.:Hirayama, B.:Takahashi, T. | Evaluation of the treatment modalities for neurosensory disturbances of the inferior alveolar nerve following retromolar bone harvesting for bone augmentation | Pain Medicine | 16 | 3 | 2015 | no outcomes of interest |
|  | Pistilli, R.:Felice, P.:Piatelli, M.:Nisii, A.:Barausse, C.:Esposito, M. | Blocks of autogenous bone versus xenografts for the rehabilitation of atrophic jaws with dental implants: preliminary data from a pilot randomised controlled trial | European Journal of Oral Implantology | 7 | 2 | 2014 | cannot distinguish VRA cases from HRA |
|  | Reissmann, D. R.:Dietze, B.:Vogeler, M.:Schmelzeisen, R.:Heydecke, G. | Impact of donor site for bone graft harvesting for dental implants on health-related and oral health-related quality of life | Clinical Oral Implants Research | 24 | 6 | 2013 | non-randomized |
|  | Rocchietta, I.:Simion, M.:Hoffmann, M.:Trisciuoglio, D.:Benigni, M.:Dahlin, C. | Vertical Bone Augmentation with an Autogenous Block or Particles in Combination with Guided Bone Regeneration: A Clinical and Histological Preliminary Study in Humans | Clinical Implant Dentistry & Related Research | 18 | 1 | 2016 | non-randomized |
|  | Heberer, S.:Ruhe, B.:Krekeler, L.:Schink, T.:Nelson, J. J.:Nelson, K. | A prospective randomized split-mouth study comparing iliac onlay grafts in atrophied edentulous patients: covered with periosteum or a bioresorbable membrane | Clinical Oral Implants Research | 20 | 3 | 2009 | only 4 months f/u |
|  | Schortinghuis, J.:Bronckers, A. L.:Stegenga, B.:Raghoebar, G. M.:de Bont, L. G. | Ultrasound to stimulate early bone formation in a distraction gap: a double blind randomised clinical pilot trial in the edentulous mandible | Archives of Oral Biology | 50 | 4 | 2005 | no outcomes of interest |
|  | Jung, R. E.:Fenner, N.:Hammerle, C. H. F.:Zitzmann, N. U. | Long-term outcome of implants placed with guided bone regeneration (GBR) using resorbable and non-resorbable membranes after 12-14years | Clinical Oral Implants Research | 24 | 10 | 2013 | not VRA cases |
|  | Jung, R. E.:Halg, G. A.:Thoma, D. S.:Hammerle, C. H. | A randomized, controlled clinical trial to evaluate a new membrane for guided bone regeneration around dental implants | Clinical Oral Implants Research | 20 | 2 | 2009 | not VRA cases |
|  | Simion, M.:Jovanovic, S. A.:Trisi, P.:Scarano, A.:Piattelli, A. | Vertical ridge augmentation around dental implants using a membrane technique and autogenous bone or allografts in humans | International Journal of Periodontics & Restorative Dentistry | 18 | 1 | 1998 | non-randomized |
|  | Smolka, W.:Eggensperger, N.:Carollo, V.:Ozdoba, C.:Iizuka, T. | Changes in the volume and density of calvarial split bone grafts after alveolar ridge augmentation | Clinical Oral Implants Research | 17 | 2 | 2006 | Hx of tumor |
|  | Spin-Neto, R.:Stavropoulos, A.:Dias Pereira, L. A.:Marcantonio, E., Jr.:Wenzel, A. | Fate of autologous and fresh-frozen allogeneic block bone grafts used for ridge augmentation. A CBCT-based analysis | Clinical Oral Implants Research | 24 | 2 | 2013 | CC study |
|  | Thor, A.:Wannfors, K.:Sennerby, L.:Rasmusson, L. | Reconstruction of the severely resorbed maxilla with autogenous bone, platelet-rich plasma, and implants: 1-year results of a controlled prospective 5-year study | Clinical Implant Dentistry & Related Research | 7 | 4 | 2005 | Pts not heathy |
|  | Torres, J.:Tamimi, F.:Alkhraisat, M. H.:Manchon, A.:Linares, R.:Prados-Frutos, J. C.:Hernandez, G.:Lopez Cabarcos, E. | Platelet-rich plasma may prevent titanium-mesh exposure in alveolar ridge augmentation with anorganic bovine bone | Journal of Clinical Periodontology | 37 | 10 | 2010 | Sinus floor elevation |
|  | Tosun, E.:Avag, C.:Baslarli, O.:Kiris, S.:Ozturk, A.:Akkocaoglu, M. | Comparison between peri-implant bone level changes of implants placed during and 3 months after iliac bone grafting | Oral Surgery, Oral Medicine, Oral Pathology and Oral Radiology | 125 | 2 | 2018 | retrospective |
|  | Uckan, S.:Oguz, Y.:Bayram, B. | Comparison of intraosseous and extraosseous alveolar distraction osteogenesis | Journal of Oral & Maxillofacial Surgery | 65 | 4 | 2007 | Hx of tumor and cleft palate |
|  | Uckan, S.:Veziroglu, F.:Dayangac, E. | Alveolar distraction osteogenesis versus autogenous onlay bone grafting for alveolar ridge augmentation: Technique, complications, and implant survival rates | Oral Surgery Oral Medicine Oral Pathology Oral Radiology & Endodontics | 106 | 4 | 2008 | Pts not heathy |
|  | Faysal, U.:Cem, S. B.:Atilla, S. | Effects of different consolidation periods on bone formation and implant success in alveolar distraction osteogenesis: a clinical study | Journal of Cranio-Maxillo-Facial Surgery | 41 | 3 | 2013 | no mention of randomization |
|  | Yerit, K. C.:Posch, M.:Guserl, U.:Turhani, D.:Schopper, C.:Wanschitz, F.:Wagner, A.:Watzinger, F.:Ewers, R. | Rehabilitation of the severely atrophied maxilla by horseshoe Le Fort I osteotomy (HLFO) | Oral Surgery Oral Medicine Oral Pathology Oral Radiology & Endodontics | 97 | 6 | 2004 | no mention of randomization- no clear inclusion crireia |
|  | Nazzal, S. Q.: Al-Dubai, M.: Mounir, R.: Ali, S.: Mounir, M. | Maxillary vertical alveolar ridge augmentation using computer-guided sandwich osteotomy technique with simultaneous implant placement versus conventional technique: A pilot study | Clinical implant dentistry and related research | 23 | 6 | 2022 | No outcome of interest |
|  | Deesricharoenkiat, N.:Jansisyanont, P.:Chuenchompoonut, V.:Mattheos, N.:Thunyakitpisal, P. | The effect of acemannan in implant placement with simultaneous guided bone regeneration in the aesthetic zone: a randomized controlled trial | International Journal of Oral and Maxillofacial Surgery | 51 | 4 | 2021 | not specific for VRA |
|  | Bettini S, Rengo C, Fiorino A, Cucchi A. | Vertical Ridge Augmentation Using Reinforced PTFE Mesh Versus Customized Titanium Mesh. Preliminary Results Of A Randomized Clinical Trial. | IAO in Milan Italy 2020. |  |  | 2020 | Only abstract available |
|  | Byun, S. H.:Kim, S. H.:Cho, S.:Lee, H.:Lim, H. K.:Lee, U. L.:Song, W.:Kim, S. J.:Kim, M. K.:Kim, J. W. | Tissue expansion improves the outcome and predictability for alveolar bone augmentation: Prospective, multicenter, randomized controlled trial | Journal of Clinical Medicine | 9 | 4 |  | Excluded following author correspondence- not randomised properly |
|  | Li, S.:Zhao, J.:Xie, Y.:Tian, T.:Zhang, T.:Cai, X. | Hard tissue stability after guided bone regeneration: a comparison between digital titanium mesh and resorbable membrane | International Journal of Oral Science | 13 | 1 | 2021 | Retrospective |
|  | Schwarz, F.:Obreja, K.:Mayer, S.:Ramanauskaite, A.:Sader, R.:Parvini, P. | Efficacy of autogenous tooth roots for a combined vertical and horizontal alveolar ridge augmentation and staged implant placement. A prospective controlled clinical study | Journal of Clinical Periodontology. | 45 | 8 | 2018 | non randomised |
|  | Brenton Lahey | Ridge augmentation comparing a cancellous block allograft to freeze dried bone particles and utilizing an acellular dermal matrix barrier membrane. | Thesis- University of Louisville |  |  | 2005 | Cases are not clearly defined |
|  | Al-Dubai, M., Mounir, R., Ali, S., Mounir, M. | Maxillary vertical alveolar ridge augmentation using sandwich osteotomy technique with simultaneous versus delayed implant placement: A proof of principle randomized clinical trial | Clinical Implant Dentistry & Related Research | 14 |  | 2022 | No outcome of interst |
|  | de Almeida Malzoni, C. M.  Goncalves, V.  Possari, J.  Junior, E. M. | The use of 3D ceramic block graft compared with autogenous block graft for rehabilitation of the atrophic maxilla: a randomized controlled clinical trial | Trials | 23 | 1 | 2022 | Protocol |
|  | Fonseca, M.  Adanez, M. H.  Pieralli, S.  Brezavscek, M.  Yilmaz, B.  Att, W. | Short Versus Regular-Length Implants to Rehabilitate Partially Edentulous Mandible: A 2-Year Prospective Split-Mouth Clinical Study | The Journal of oral implantology | 48 | 4 | 2022 | Not RCT |
|  | Gallo, P.  Diaz-Baez, D.  Perdomo, S.  Aloise, A. C.  Tattan, M.  Saleh, M. H. A.  Pelegrine, A. A.  Ravida, A.  Wang, H. L. | Comparative analysis of two biomaterials mixed with autogenous bone graft for vertical ridge augmentation: A histomorphometric study in humans | Clinical Implant Dentistry & Related Research | 24 | 5 | 2022 | Not RCT |
|  | Guida, L.  Annunziata, M.  Esposito, U.  Sirignano, M.  Torrisi, P.  Cecchinato, D. | 6-mm implants for the full-arch rehabilitation of edentulous mandibles: 5-year results from a multicenter randomized controlled trial | Journal of Clinical Periodontology | 49(Supplement 23) |  | 2022 | Only abstract available |
|  | Jung, R. E.  Kovacs, M. N.  Thoma, D. S.  Hammerle, C. H. F. | Informative title: Guided bone regeneration with and without rhBMP-2: 17-year results of a randomized controlled clinical trial | Clinical Oral Implants Research | 33 | 3 | 2022 | Not VRA cases |
|  | Li, L.  Gao, H.  Ji, P.  Huang, Y.  Wang, C. | Assessment of Customized Alveolar Bone Augmentation Using Titanium Scaffolds vs Polyetheretherketone (PEEK) Scaffolds: A Comparative Study Based on 3D Printing Technology | ACS Biomaterials Science and Engineering. |  |  | 2022 | Animals study |
|  | Longhi, B.  Gibello, U.  Bettini, S.  Corinaldesi, G.  Cucchi, A. | RCT preliminary data on vertical bone gain, bone density, pseudo-periosteum and implant stability after vertical ridge augmentation | Journal of Clinical Periodontology | 49(Supplement 23) |  | 2022 | Only abstract available |
|  | Markovic, L.  Smojver, I.  Vuletic, M.  Morelato, L.  Brzovic Rajic, V.  Gabric, D. | Autologous dentin graft for alveolar ridge augmentation - 2 years follow-up of clinical study | Journal of Clinical Periodontology | 49(Supplement 23) |  | 2022 | Only abstract available |
|  | Naujokat, H.  Loger, K.  Gulses, A.  Florke, C.  Acil, Y.  Wiltfang, J. | Effect of enriched bone-marrow aspirates on the dimensional stability of cortico-cancellous iliac bone grafts in alveolar ridge augmentation | International Journal of Implant Dentistry | 8 | 1 | 2022 | Not RCT |
|  | Schwarz, F.  Obreja, K.  Mayer, S.  Ramanauskaite, A.  Sader, R.  Parvini, P. | Efficacy of autogenous tooth roots for a combined vertical and horizontal alveolar ridge augmentation and staged implant placement. A prospective controlled clinical study | Journal of Clinical Periodontology | 49 | 5 | 2022 | Not RCT |
|  | Wang, M.  Li, Y.  Su, Z.  Mo, A. | Clinical and radiographic outcomes of customized allogeneic bone block versus autogenous bone block for ridge augmentation: 6 Month results of a randomized controlled clinical trial | Journal of Clinical Periodontology | 23 |  | 2022 | Not all VRA cases |
|  | Y. E. S. Fekry  N. R. Mahmoud | Vertical ridge augmentation of atrophic posterior mandible with corticocancellous onlay symphysis graft versus sandwich technique: clinical and radiographic analysis | Odontolog | 111 | 4 | 2023 | Incomplete data |
|  | H. Koo,  J. Hwang,  B. J. Choi,  J. W. Lee,  J. Y. Ohe  J. Jung | Comparison of vertical bone resorption following various types of autologous block bone grafts | Maxillofacial Plastic & Reconstructive Surgery | 45 | 1 | 2023 | Non-randomised |
|  | A. E. D. Ragab Mahmoud,  S. M. Yassin,  S. A. Hassan  H. S. Abdelmoneim | Vertical alveolar bone augmentation of atrophied posterior mandibular regions with simultaneous dental implant placement using allogeneic bone rings vs autogenous bone rings: a randomized controlled clinical trial | Quintessence International | 55 | 3 | 2024 | Simultaneous approach |
|  | N. H. Kim,  B. E. Yang,  S. W. On,  I. J. Kwon,  K. M. Ahn,  J. H. Lee, et al. | Customized three-dimensional printed ceramic bone grafts for osseous defects: a prospective randomized study | Scientific Reports | 14 | 1 | 2024 | PBR% not reported |
|  | C. Mertens,  C. Busch,  K. Goldenbaum,  O. Ristow, J. Hoffmann, H. L. Wang, et al. | Full block or split block?-Comparison of two different autogenous block grafting techniques for alveolar ridge reconstruction | Clinical implant dentistry and related research | 25 | 6 |  | Retrospective |

**Appendix 1.4:** **Risk of bias within studies**


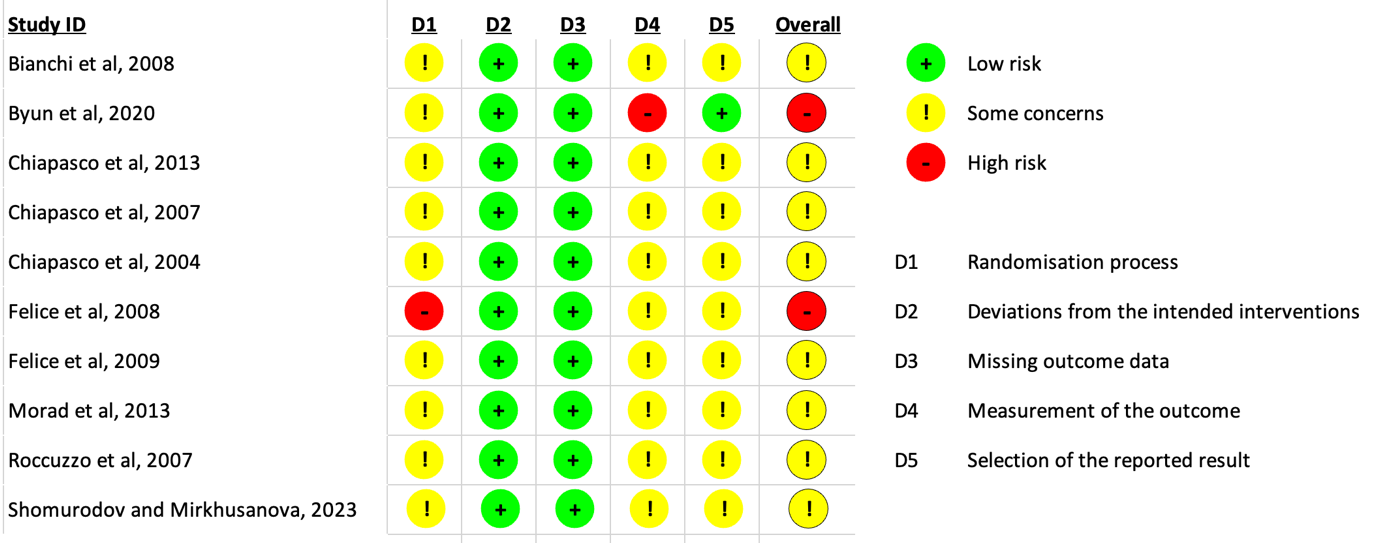


**Appendix 2.4: Node-splitting models**

|  | **comparison** | **p.value** | **CrI** |
| --- | --- | --- | --- |
| **1** | d.DO.Inlay | 0.135300 | NA |
| **2** | -> direct | NA | -3.1 (-34., 27.) |
| **3** | -> indirect | NA | -42. (-96., 11.) |
| **4** | -> network | NA | -11. (-45., 20.) |
| **5** | d.DO.Onlay | 0.133900 | NA |
| **6** | -> direct | NA | 6.0 (-25., 37.) |
| **7** | -> indirect | NA | 45. (-8.6, 98.) |
| **8** | -> network | NA | 15. (-17., 48.) |
| **9** | d.Inlay.OnlayBarrier | 0.140750 | NA |
| **10** | -> direct | NA | 30. (-1.6, 61.) |
| **11** | -> indirect | NA | -8.8 (-62., 45.) |
| **12** | -> network | NA | 20. (-13., 53.) |
| **13** | d.Onlay.OnlayBarrier | 0.135075 | NA |
| **14** | -> direct | NA | -18. (-50., 14.) |
| **15** | -> indirect | NA | 21. (-32., 74.) |
| **16** | -> network | NA | -6.9 (-41., 25.) |

**References:**

Buti J, Glenny A-M, Worthington HV, Nieri M, Baccini M. 2011. Network meta-analysis of randomised controlled trials: Direct and indirect treatment comparisons. Eur J Oral Implantol. 4(1):55-62.

Riley RD, Higgins JP, Deeks JJ. 2011. Interpretation of random effects meta-analyses. BMJ. 342.

Rücker G, Schwarzer G, Carpenter JR, Schumacher M. 2008. Undue reliance on i 2 in assessing heterogeneity may mislead. BMC Med Res Methodol. 8:1-9.
